# Crystal structures of (aceto­nitrile-κ*N*)tris­(pyridine-4-thio­amide-κ*N*)bis­(thio­cyanate-κ*N*)cobalt(II) aceto­nitrile disolvate and tetra­kis­(pyridine-4-thio­amide-κ*N*)bis­(thio­cyanate-κ*N*)nickel(II) methanol penta­solvate

**DOI:** 10.1107/S2056989018007612

**Published:** 2018-06-12

**Authors:** Tristan Neumann, Inke Jess, Christian Näther

**Affiliations:** a Institut für Anorganische Chemie, Christian-Albrechts-Universität Kiel, Max-Eyth Str. 2, D-24118 Kiel, Germany

**Keywords:** crystal structure, discrete complexes, thio­cyanate, hydrogen bonding, cobalt, nickel

## Abstract

Reaction of Co(NCS)_2_ or Ni(NCS)_2_ with pyridine-4-thio­amide in different solvents led to the formation of two compounds with composition [Co(NCS)_2_(C_2_H_3_N)(C_6_H_6_N_2_S)_3_]·2CH_3_CN (**1**) and [Ni(NCS)_2_(C_6_H_6_N_2_S)_4_]·5CH_3_OH (**2**), respectively. The asymmetric unit of compound **1** consists of one cobalt(II) cation, two thio­cyanate anions, three pyridine-4-thio­amide ligands, one coordinating and two solvate aceto­nitrile mol­ecules. One of the two aceto­nitrile solvate mol­ecules is disordered over two sets of sites in a 0.62:0.38 ratio. The asymmetric unit of compound **2** comprises of one nickel(II) cation, two thio­cyanate anions, four N-bonding pyridine-4-thio­amide ligands and five methanol solvate mol­ecules. In compound **1**, the cobalt(II) cations are octa­hedrally coordinated into discrete complexes by two terminal N-bonding thio­cyanate anions, the N atoms of three pyridine-4-thio­amide ligands and one aceto­nitrile mol­ecule. Additional aceto­nitrile solvate mol­ecules are located between the complexes,. The complexes and solvate mol­ecules are linked *via* inter­molecular hydrogen bonding into a three-dimensional framework. In compound **2**, the nickel(II) cations are likewise octa­hedrally coordinated by two terminal N-bonded thio­cyanate anions and four N-bonding pyridine-4-thio­amide ligands into discrete complexes. From their arrangement cavities are formed, in which the methanol solvate mol­ecules are located. Again, the complexes and solvate mol­ecules are linked into a three-dimensional framework by inter­molecular hydrogen bonding.

## Abstract 2

The crystal structures of the title compounds consist of discrete octa­hedral complexes that are linked by inter­molecular hydrogen bonding between the complexes and additional solvate mol­ecules into three-dimensional network structures.

## Chemical context   

For several years we have been inter­ested in the structural, thermal and magnetic properties of coordination compounds and polymers based on transition metal thio- and seleno­cyanates (Wöhlert *et al.*, 2013*a* ▸, 2014*a* ▸). In contrast to other three-atomic ligands such as, for example azides, these ligands show a more versatile coordination behaviour, including a terminal coordination and a number of different bridging modes. Therefore they are of inter­est from a structural point of view (Massoud *et al.*, 2013 ▸; Mousavi *et al.*, 2012 ▸; Prananto *et al.*, 2017 ▸; Kabešová *et al.*, 1995 ▸; Palion-Gazda *et al.*, 2017 ▸). Moreover, if paramagnetic metal cations are linked by these anionic ligands into chains or layers, cooperative magnetic phenomena can be expected. Hence the rational synthesis of such compounds is in the focus of our investigations (Palion-Gazda *et al.*, 2015 ▸; Wöhlert *et al.*, 2013*a* ▸). In this context, compounds of special inter­est include those in which the metal cations are linked by pairs of anionic ligands into linear chains because they can exhibit one-dimensional or three-dimensional ferromagnetic ordering, as shown recently for a number of compounds derived from Co(NCS)_2_ (Rams *et al.*, 2017*a* ▸,*b* ▸; Wöhlert *et al.* 2012 ▸, 2013*b* ▸, 2014*b* ▸; Werner *et al.*, 2015 ▸). Unfortunately, the paramagnetic metal cations Co^II^ or Ni^II^ are less chalcophilic and therefore do not form compounds with polymeric structures from solutions, but with discrete complexes instead. In the majority of cases, these cations are octa­hedrally coordinated by two anionic ligands and four monodentate N-donor co-ligands. However, if such complexes are heated, they frequently decompose in discrete steps, forming new compounds as inter­mediates in which the metal cations are linked into one- or two-dimensional network structures. This is the reason why we are also inter­ested in such simple complexes or their solvates (Suckert *et al.*, 2017 ▸).
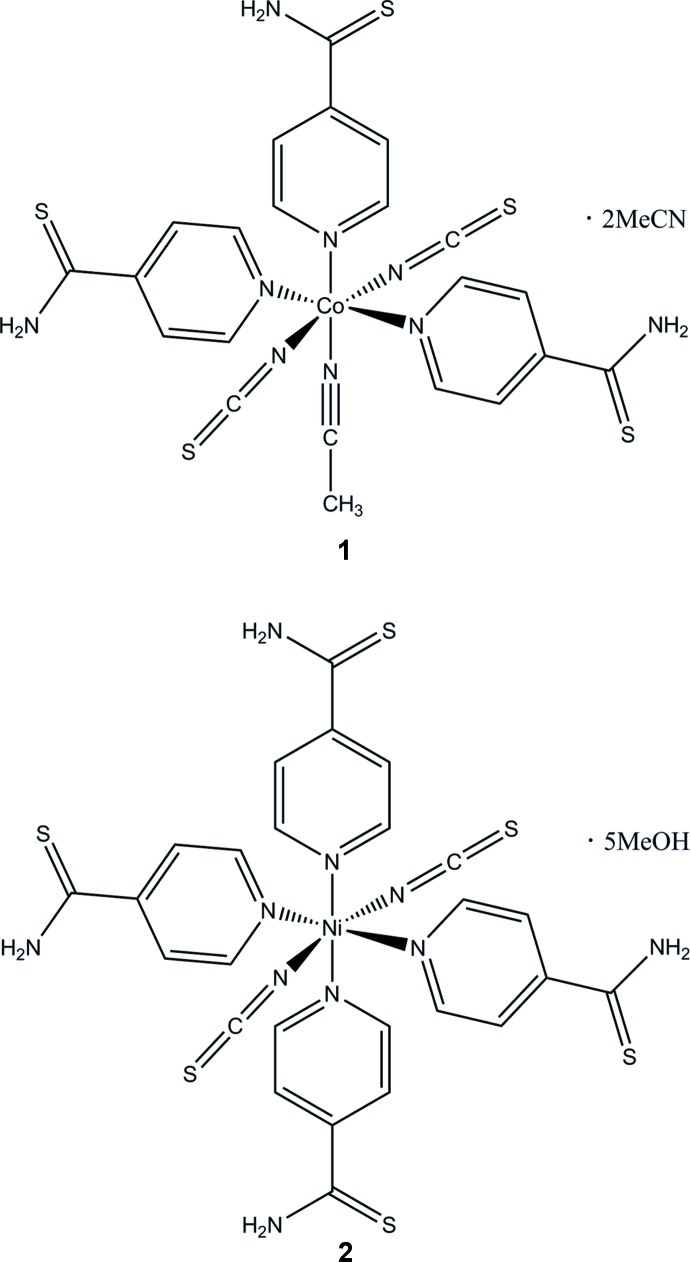


In the course of our project we became inter­ested in the monodentate ligand pyridine-4-thio­amide, which might be able to link *M*(NCS)_2_ chains (*M* = Co, Ni) into layers by inter­molecular N—H⋯S hydrogen bonding. For example, this motif is observed in the crystal structure of the pure ligand (Colleter & Gadret, 1967 ▸; Eccles *et al.*, 2014 ▸). Moreover, one compound derived from Cd(NCS)_2_ is known in which the metal cations are linked by pairs of anionic ligands into chains (Neumann *et al.*, 2016 ▸). Therefore we attempted in the synthesis of discrete precursor complexes or solvates in which the anionic ligands are only terminal N-bonding to transform them subsequently into the desired chain compounds by thermal annealing. Unfortunately, no pure samples could be obtained (Neumann *et al.*, 2017 ▸,2018 ▸). In the course of this work we obtained two additional compounds from aceto­nitrile or methanol solution, *viz*. [Co(NCS)_2_(C_6_H_6_N_2_S)_3_(C_2_H_3_N)]·2C_2_H_3_N (**1**) and [Ni(NCS)_2_(C_6_H_6_N_2_S)_4_]·5CH_3_OH (**2**), for which the CN stretching vibration is observed at 2081 cm^−1^ (**1**) and 2101 cm^−1^ (**2**), respectively. As a consequence, their structures should consist of discrete complexes with terminal N-bonded thio­cyanate anions and additional solvate mol­ecules, even if these wave numbers are at the borderline of those expected for the desired bridging anionic ligands. To check if our assumption can be verified, we have performed single-crystal structure determinations of **1** and **2** and report the results in this communication.

## Structural commentary   

Unfortunately, **1** and **2** could not be prepared as pure phases and were either contaminated with additional unknown crystalline phases or, if an excess of pyridine-4-thio­amide was used, with this less soluble ligand. Therefore, no further investigations regarding physical properties were performed.

**Figure 1:**
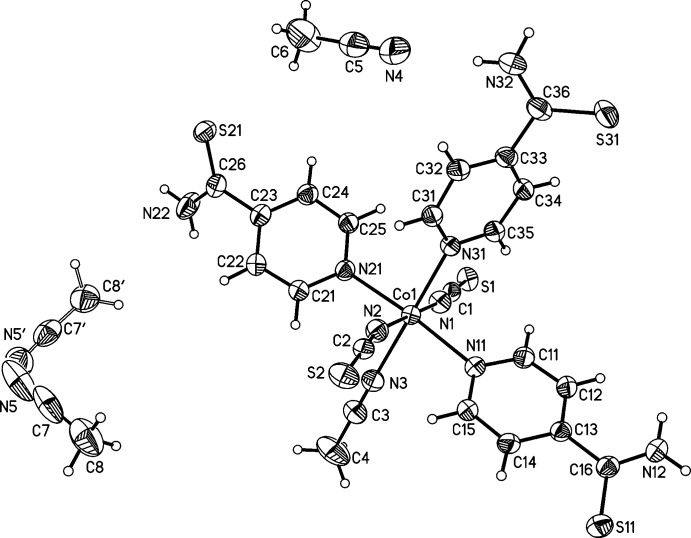
View of the asymmetric unit of compound **1** with atom labelling and displacement ellipsoids drawn at the 50% probability level. The disordered aceto­nitrile solvent mol­ecule is shown with both orientations.

The asymmetric unit of compound **1** consists of one cobalt(II) cation, two thio­cyanate anions, three pyridine-4-thio­amide ligands and three aceto­nitrile mol­ecules. One of the two aceto­nitrile solvate mol­ecules is disordered over two sets of sites in a refined ratio of 0.62:0.38. The Co^II^ cation is octa­hedrally coordinated by two terminal N-bonding thio­cyanate anions, an acetonitrile molecule and the pyridine N atoms of three pyridine-4-thio­amide ligands into a discrete complex with the same ligand types *trans*-positioned to each other (Fig. 1 ▸). The Co—N bond lengths to the thio­cyanate anions are significantly shorter than those to the pyridine N atoms (Table 1 ▸), in agreement with values for similar structures (Goodgame *et al.*, 2003 ▸; Prananto *et al.*, 2017 ▸). The bond angles deviate from ideal values, showing that the octa­hedra are slightly distorted (Table 1 ▸).

**Figure 2:**
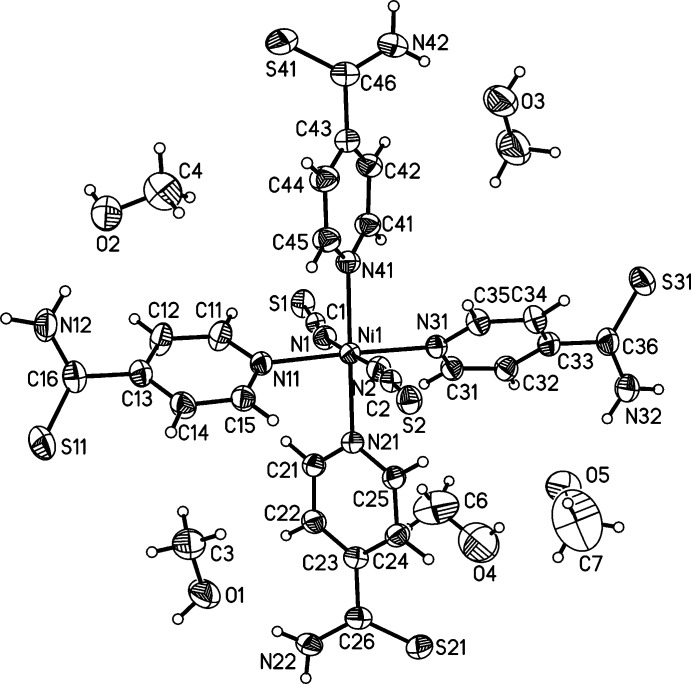
View of the asymmetric unit of compound **2** with atom labelling and displacement ellipsoids drawn at the 50% probability level.

The asymmetric unit of compound **2** comprises of one nickel(II) cation, two thio­cyanate anions, four N-bonded pyridine-4-thio­amide ligands and five methanol solvate mol­ecules (Fig. 2 ▸). The Ni^II^ cation is also octa­hedrally coordinated by N atoms, but in this case by four pyridine-4-thio­amide ligands and two terminal thio­cyanate anions. Bond lengths and angles (Table 2 ▸) are comparable to those in the structure of compound **1**, but the NiN_6_ octa­hedron is less distorted than the CoN_6_ octa­hedron. It is noted that in both structures the pyridine-4-thio­amide ligands are not planar. The thio­amide groups are rotated differently out of the pyridine ring plane, with dihedral angles in the range 5.3 (2)–54.5 (2)° for **1** and 40.7 (2)–47.2 (2)° for **2**.

## Supra­molecular features   

**Figure 3:**
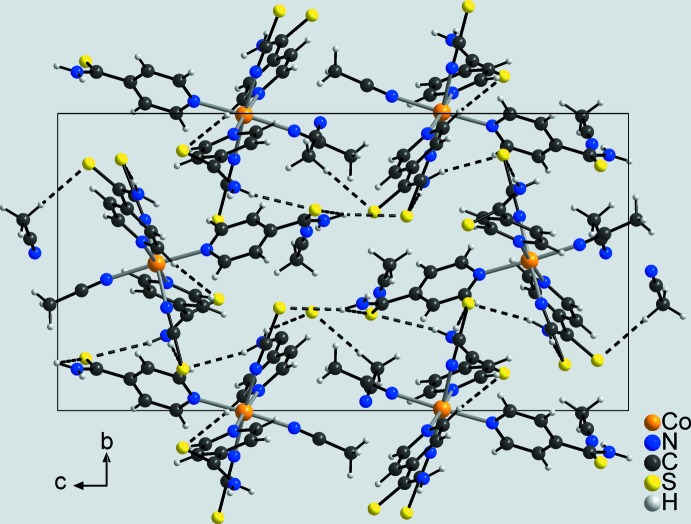
Crystal structure of compound **1** in a view along the *a* axis. Inter­molecular hydrogen bonding is shown as dashed lines.

In the crystal structure of compound **1**, the discrete complexes are linked by inter­molecular N—H⋯S hydrogen bonding between the H atoms of the amino groups and the S atoms of the thio­cyanate anions or the pyridine-4-thio­amide ligands into a three-dimensional framework (Fig. 3 ▸, Table 3 ▸). The complexes are arranged in such a way that cavities are formed in which additional aceto­nitrile mol­ecules are embedded. These solvate mol­ecules are linked together *via* C—H⋯N inter­actions between the methyl H atoms and the N atom of the aceto­nitrile mol­ecules, but are also connected to the metal complexes by inter­molecular C—H⋯N and C—H⋯S inter­actions.

**Figure 4:**
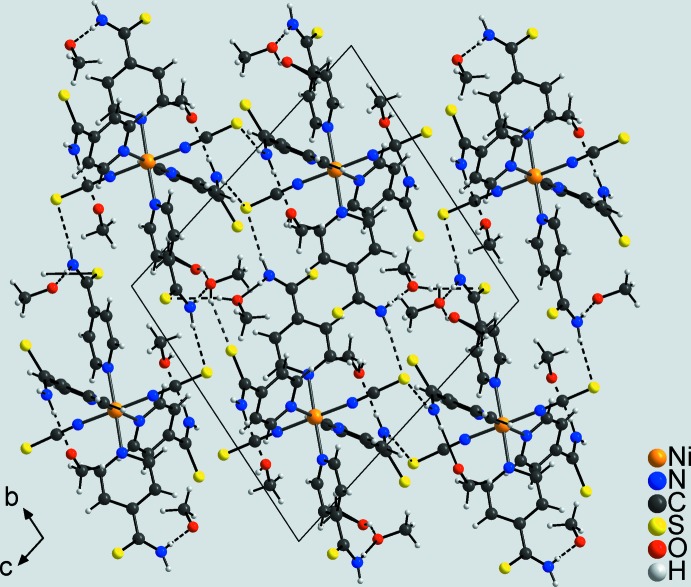
Crystal structure of compound **2** in a view along the *a* axis. Inter­molecular hydrogen bonding is shown as dashed lines.

In the crystal structure of compound **2**, a variety of different hydrogen-bonding inter­actions is observed in which the methanol solvate mol­ecules act both as acceptor and donor groups. Like in compound **1**, the complexes are connected into a three-dimensional framework by inter­molecular N—H⋯S hydrogen bonding between the H atoms of the amino groups and the S atoms of the thio­cyanate anions. Again, cavities are formed that host the methanol solvate mol­ecules. These mol­ecules are linked by inter­molecular O—H⋯O hydrogen bonding to other methanol mol­ecules, but are also connected to the complexes by N—H⋯O and O—H⋯S hydrogen bonds to the amino groups and the S atoms of the pyridine-4-thio­amide ligands and to the thio­cyanate S atoms (Fig. 4 ▸, Table 4 ▸). Finally, C—H⋯N and C—H⋯S inter­actions consolidate the packing of the mol­ecules in the structure.

## Database survey   

There are only two cobalt thio­cyanate derivatives with additional pyridine-4-thio­amide ligands reported in the Cambridge Structure Database (Version 5.39, last update February 2018; Groom *et al.*, 2016 ▸). In tetra­kis­(pyridine-4-carbo­thio­amide-κ*N* ^1^)bis-(thio­cyanato-κ*N*)cobalt(II) methanol monosolvate and tetra­kis­(pyridine-4-carbo­thio­amide-κ*N* ^1^)bis-(thio­cyanato-κ*N*)cobalt(II) monohydrate, the Co^II^ cations are octa­hedrally coordinated by four pyridine-4-carbo­thio­amide ligands and two thio­cyanate anions, with the different types of solvent mol­ecules being located in cavities of the structure (Neumann *et al.*, 2017 ▸, 2018 ▸). In Zn(NCS)_2_(pyridine-4-thio­amide)_2_, the Zn^II^ cations are tetra­hedrally coordinated by two thio­cyanate anions and two pyridine-4-thio­amide ligands (Neumann *et al.*, 2018 ▸). In addition there is one compound with cadmium, in which the Cd^II^ cations are octa­hedrally coordinated by two terminal N-bonded pyridine­thio­amide ligands and four thio­cyanate anions and linked by pairs of anionic ligands into linear chains (Neumann *et al.*, 2016 ▸). Alongside the structure of the pure pyridine-4-thio­amide ligand (Colleter & Gadret, 1967 ▸; Eccles *et al.*, 2014 ▸), its protonated form with iodide as counter-anion was reported by Shotonwa & Boeré (2014 ▸).

## Synthesis and crystallization   

Co(NCS)_2_ and pyridine-4-thio­amide were purchased from Alfa Aesar. Ni(NCS)_2_ was prepared by the reaction of equimolar amounts of Ba(SCN)_2_·3H_2_O with NiSO_4_·6H_2_O in water. The colourless precipitate of BaSO_4_ was filtered off and the resulting clear solution was evaporated until complete dryness. The purity of Ni(NCS)_2_ was checked by X-ray powder diffraction measurements.

Crystals of compound **1** were obtained by the reaction of 8.8 mg Co(NCS)_2_ (0.05 mmol) with 13.8 mg pyridine-4-thio­amide (0.1 mmol) in 1 ml aceto­nitrile. The reaction mixture was left to stand at room-temperature, leading to a few crystals of the title compound suitable for single-crystal X-ray diffraction.

For the synthesis of compound **2**, 8.8 mg Ni(NCS)_2_ (0.05 mmol) were reacted with 27.6 mg pyridine-4-thio­amide (0.2 mmol) in 3.0 ml methanol. The mixture was heated to the boiling temperature of methanol and then slowly cooled down, leading to the formation of a few crystals suitable for single-crystal X-ray diffraction.

All reaction batches were contaminated with additional crystalline phases that are unknown. If an excess of pyridine-4-thio­amide was used to shift the equillibria in the directions of the discrete complexes with only coordinating pyridine-4-thio­amide ligands, the batches were always contaminated with this organic ligand because it is poorly soluble in the used solvents.

IR spectra of manually selected crystals are included for both compounds in the supporting information.

## Refinement   

Crystal data, data collection and structure refinement details are summarized in Table 5 ▸. The C—H hydrogen atoms were positioned with idealized geometry (C—H = 0.95–0.98 Å; methyl H atoms were allowed to rotate but not to tip) and were refined with *U*_iso_(H) = 1.2*U*_eq_(C) (1.5 for methyl and hydroxyl H atoms) using a riding model. The N—H hydrogen atoms were located in a difference-Fourier map, their bond lengths set to ideal values (N—H = 0.88 Å) and refined with *U*_iso_(H) = 1.5*U*_eq_(N) using a riding model. In **1**, one of the two crystallographically independent aceto­nitrile solvent mol­ecules is disordered over two sets of sites and was refined using a split model with restraints [SAME in *SHELXL* (Sheldrick, 2015 ▸)], leading to a ratio of 0.62:0.38 for the two orientations (fixed at the final stage of refinement).

## Supplementary Material

Crystal structure: contains datablock(s) Compound1, Compound2. DOI: 10.1107/S2056989018007612/wm5443sup1.cif

Structure factors: contains datablock(s) Compound1. DOI: 10.1107/S2056989018007612/wm5443Compound1sup2.hkl

Structure factors: contains datablock(s) Compound2. DOI: 10.1107/S2056989018007612/wm5443Compound2sup3.hkl

Figs. S1 and S2. IR-Data for compounds 1 and 2. DOI: 10.1107/S2056989018007612/wm5443sup4.pdf

CCDC references: 1844699, 1844698

Additional supporting information:  crystallographic information; 3D view; checkCIF report

## Figures and Tables

**Table 1:** Selected geometric parameters (Å, °) for **1** [Chem scheme1]

Co1—N1	2.0650 (16)	Co1—N31	2.1785 (14)
Co1—N2	2.0720 (16)	Co1—N3	2.1950 (15)
Co1—N21	2.1666 (15)	Co1—N11	2.2032 (15)
			
N1—Co1—N2	177.65 (6)	N21—Co1—N3	88.82 (6)
N1—Co1—N21	91.08 (6)	N31—Co1—N3	177.72 (6)
N2—Co1—N21	88.02 (6)	N1—Co1—N11	92.70 (6)
N1—Co1—N31	89.52 (6)	N2—Co1—N11	88.06 (6)
N2—Co1—N31	92.66 (6)	N21—Co1—N11	174.69 (5)
N21—Co1—N31	90.26 (6)	N31—Co1—N11	93.50 (5)
N1—Co1—N3	88.41 (6)	N3—Co1—N11	87.56 (6)
N2—Co1—N3	89.39 (6)		

**Table 2:** Selected geometric parameters (Å, °) for **2** [Chem scheme1]

Ni1—N1	2.0435 (18)	Ni1—N31	2.1250 (17)
Ni1—N2	2.0526 (18)	Ni1—N41	2.1262 (17)
Ni1—N21	2.1157 (16)	Ni1—N11	2.1316 (17)
			
N1—Ni1—N2	178.69 (7)	N21—Ni1—N41	179.30 (7)
N1—Ni1—N21	90.21 (7)	N31—Ni1—N41	90.22 (7)
N2—Ni1—N21	90.67 (7)	N1—Ni1—N11	91.28 (7)
N1—Ni1—N31	89.06 (7)	N2—Ni1—N11	89.71 (7)
N2—Ni1—N31	89.98 (7)	N21—Ni1—N11	88.91 (6)
N21—Ni1—N31	89.16 (6)	N31—Ni1—N11	178.04 (6)
N1—Ni1—N41	89.46 (7)	N41—Ni1—N11	91.72 (7)
N2—Ni1—N41	89.65 (7)		

**Table 3:** Hydrogen-bond geometry (Å, °) for **1** [Chem scheme1]

*D*—H⋯*A*	*D*—H	H⋯*A*	*D*⋯*A*	*D*—H⋯*A*
C4—H4*C*⋯N5′^i^	0.98	2.37	3.081 (12)	129
C6—H6*C*⋯S31^ii^	0.98	3.02	3.901 (3)	150
C11—H11⋯S21^i^	0.95	2.83	3.6556 (18)	146
C12—H12⋯S1^iii^	0.95	3.01	3.8491 (18)	148
N12—H1*N*⋯S1^iii^	0.88	2.66	3.5097 (17)	163
N12—H2*N*⋯S2^i^	0.88	2.71	3.5731 (17)	167
C21—H21⋯N3	0.95	2.63	3.134 (3)	114
C22—H22⋯N5^iv^	0.95	2.50	3.384 (7)	154
C25—H25⋯S2^v^	0.95	2.91	3.7172 (18)	144
N22—H3*N*⋯S1^vi^	0.88	2.59	3.4715 (19)	179
N22—H4*N*⋯S31^v^	0.88	2.87	3.729 (2)	167
C34—H34⋯S11^vii^	0.95	2.98	3.7698 (19)	142
C35—H35⋯N1	0.95	2.56	3.094 (2)	116
C35—H35⋯S21^i^	0.95	2.95	3.7301 (19)	140
N32—H5*N*⋯S2^viii^	0.88	2.74	3.5390 (18)	152
N32—H6*N*⋯N4	0.88	2.10	2.951 (3)	164
C8—H8*B*⋯S11^vi^	0.98	2.76	3.728 (19)	172
C8′—H8*D*⋯N5′^iv^	0.98	2.46	3.26 (2)	140
C8′—H8*F*⋯S11^ix^	0.98	2.88	3.65 (3)	137

Symmetry codes: (i) 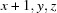; (ii) 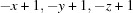; (iii) 
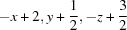
; (iv) 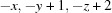; (v) 
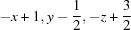
; (vi) 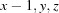; (vii) 
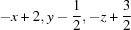
; (viii) 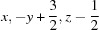; (ix) 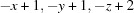.

**Table 4:** Hydrogen-bond geometry (Å, °) for **2** [Chem scheme1]

*D*—H⋯*A*	*D*—H	H⋯*A*	*D*⋯*A*	*D*—H⋯*A*
O1—H1⋯S2^i^	0.84	2.88	3.409 (2)	123
O1—H1⋯S11^ii^	0.84	2.92	3.578 (2)	137
O2—H2⋯S31^iii^	0.84	2.62	3.413 (3)	157
O3—H3⋯S1^iv^	0.84	2.77	3.427 (2)	136
O3—H3⋯S31^v^	0.84	2.93	3.576 (2)	135
C5—H5*A*⋯S31^v^	0.98	3.03	3.632 (4)	121
O4—H4⋯O5	0.84	1.92	2.708 (5)	156
C6—H6*B*⋯S31^vi^	0.98	2.73	3.566 (4)	143
O5—H5⋯S41^vii^	0.84	2.51	3.216 (3)	142
C7—H7*A*⋯S41^vii^	0.98	2.93	3.528 (5)	121
C11—H11⋯N1	0.95	2.52	3.063 (3)	117
C11—H11⋯S1^viii^	0.95	2.73	3.442 (2)	133
C12—H12⋯S1^viii^	0.95	2.96	3.542 (2)	121
C15—H15⋯N2	0.95	2.61	3.097 (3)	113
N12—H1*N*⋯O2	0.88	2.02	2.898 (3)	177
N12—H2*N*⋯S2^ix^	0.88	2.58	3.446 (2)	171
C21—H21⋯N1	0.95	2.65	3.109 (3)	110
C25—H25⋯N2	0.95	2.66	3.122 (3)	111
C25—H25⋯S2^x^	0.95	2.94	3.846 (2)	159
N22—H4*N*⋯S2^xi^	0.88	2.64	3.4939 (19)	163
N22—H3*N*⋯O1	0.88	2.10	2.978 (3)	174
N32—H5*N*⋯O5	0.88	1.95	2.833 (3)	180
N32—H6*N*⋯S1^vi^	0.88	2.64	3.478 (2)	159
C45—H45⋯S11^ix^	0.95	2.89	3.673 (2)	141
N42—H7*N*⋯O3	0.88	2.08	2.957 (3)	173
N42—H8*N*⋯S1^xii^	0.88	2.88	3.749 (2)	169

Symmetry codes: (i) 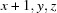; (ii) 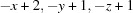; (iii) 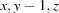; (iv) 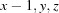; (v) 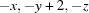; (vi) 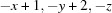; (vii) 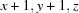; (viii) 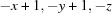; (ix) 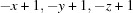; (x) 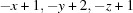; (xi) 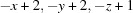; (xii) 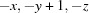.

**Table 5:** Experimental details

	**1**	**2**
Crystal data		
Chemical formula	[Co(NCS)_2_(C_2_H_3_N)(C_6_H_6_N_2_S)_3_]·2C_2_H_3_N	[Ni(NCS)_2_(C_6_H_6_N_2_S)_4_]·5CH_4_O
*M*_r_	712.81	887.83
Crystal system, space group	Monoclinic, *P*2_1_/*c*	Triclinic, *P* 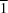
Temperature (K)	200	200
*a*, *b*, *c* (Å)	11.3566 (4), 12.3251 (2), 23.7557 (8)	10.4520 (3), 14.5934 (4), 15.0580 (5)
α, β, γ (°)	90, 93.273 (3), 90	101.553 (2), 97.105 (2), 106.417 (2)
*V* (Å^3^)	3319.69 (17)	2118.43 (11)
*Z*	4	2
Radiation type	Mo *K*α	Mo *K*α
μ (mm^−1^)	0.87	0.80
Crystal size (mm)	0.12 × 0.10 × 0.08	0.30 × 0.18 × 0.10

Data collection		
Diffractometer	Stoe IPDS2	Stoe IPDS2
Absorption correction	–	Numerical (*X-RED* and *X-SHAPE*; Stoe, 2008 ▸)
*T*_min_, *T*_max_	–	0.622, 0.889
No. of measured, independent and observed [*I* > 2σ(*I*)] reflections	25043, 7218, 6002	30865, 9253, 7895
*R*_int_	0.027	0.031
(sin θ/λ)_max_ (Å^−1^)	0.639	0.639

Refinement		
*R*[*F* ^2^ > 2σ(*F* ^2^)], *wR*(*F* ^2^), *S*	0.032, 0.080, 1.04	0.039, 0.109, 1.06
No. of reflections	7218	9253
No. of parameters	419	486
No. of restraints	9	0
H-atom treatment	H-atom parameters constrained	H-atom parameters constrained
Δρ_max_, Δρ_min_ (e Å^−3^)	0.34, −0.33	0.62, −0.57

Computer programs: *X-AREA* (Stoe, 2008 ▸), *SHELXS97* (Sheldrick, 2008 ▸), *SHELXL2014* (Sheldrick, 2015 ▸), *DIAMOND (Brandenburg, 1990 ▸) and *publCIF* (Westrip, 2010 ▸).*

## supplementary crystallographic information

### (Acetonitrile-κN)tris(pyridine-4-thioamide-κN)bis(thiocyanato-κN)cobalt(II) acetonitrile disolvate (Compound1) Crystal data

**Table:**

[Co(NCS)_2_(C_2_H_3_N)(C_6_H_6_N_2_S)_3_]·2C_2_H_3_N	*F*(000) = 1468
*M**_r_* = 712.81	*D*_x_ = 1.426 Mg m^−^^3^
Monoclinic, *P*2_1_/*c*	Mo *K*α radiation, λ = 0.71073 Å
*a* = 11.3566 (4) Å	Cell parameters from 25043 reflections
*b* = 12.3251 (2) Å	θ = 1.7–27.0°
*c* = 23.7557 (8) Å	µ = 0.87 mm^−^^1^
β = 93.273 (3)°	*T* = 200 K
*V* = 3319.69 (17) Å^3^	Block, brown
*Z* = 4	0.12 × 0.10 × 0.08 mm

### (Acetonitrile-κN)tris(pyridine-4-thioamide-κN)bis(thiocyanato-κN)cobalt(II) acetonitrile disolvate (Compound1) Data collection

**Table:**

STOE IPDS-2 diffractometer	*R*_int_ = 0.027
ω scans	θ_max_ = 27.0°, θ_min_ = 1.7°
25043 measured reflections	*h* = −14→14
7218 independent reflections	*k* = −14→15
6002 reflections with *I* > 2σ(*I*)	*l* = −30→30

### (Acetonitrile-κN)tris(pyridine-4-thioamide-κN)bis(thiocyanato-κN)cobalt(II) acetonitrile disolvate (Compound1) Refinement

**Table:**

Refinement on *F*^2^	9 restraints
Least-squares matrix: full	Hydrogen site location: mixed
*R*[*F*^2^ > 2σ(*F*^2^)] = 0.032	H-atom parameters constrained
*wR*(*F*^2^) = 0.080	*w* = 1/[σ^2^(*F*_o_^2^) + (0.0441*P*)^2^ + 0.5907*P*] where *P* = (*F*_o_^2^ + 2*F*_c_^2^)/3
*S* = 1.04	(Δ/σ)_max_ = 0.002
7218 reflections	Δρ_max_ = 0.34 e Å^−^^3^
419 parameters	Δρ_min_ = −0.33 e Å^−^^3^

### (Acetonitrile-κN)tris(pyridine-4-thioamide-κN)bis(thiocyanato-κN)cobalt(II) acetonitrile disolvate (Compound1) Special details

**Table:**

Geometry. All esds (except the esd in the dihedral angle between two l.s. planes) are estimated using the full covariance matrix. The cell esds are taken into account individually in the estimation of esds in distances, angles and torsion angles; correlations between esds in cell parameters are only used when they are defined by crystal symmetry. An approximate (isotropic) treatment of cell esds is used for estimating esds involving l.s. planes.

### (Acetonitrile-κN)tris(pyridine-4-thioamide-κN)bis(thiocyanato-κN)cobalt(II) acetonitrile disolvate (Compound1) Fractional atomic coordinates and isotropic or equivalent isotropic displacement parameters (Å^2^)

**Table:**

	*x*	*y*	*z*	*U*_iso_*/*U*_eq_	Occ. (<1)
Co1	0.62828 (2)	0.49502 (2)	0.82672 (2)	0.02661 (7)	
N1	0.70292 (15)	0.34698 (13)	0.80907 (7)	0.0359 (3)	
C1	0.73383 (16)	0.26112 (15)	0.79702 (7)	0.0307 (4)	
S1	0.77894 (5)	0.13954 (4)	0.78062 (2)	0.03821 (11)	
N2	0.55051 (14)	0.64072 (13)	0.84732 (6)	0.0343 (3)	
C2	0.52817 (15)	0.72538 (15)	0.86457 (7)	0.0295 (4)	
S2	0.49682 (5)	0.84533 (4)	0.88860 (2)	0.04202 (12)	
N3	0.61781 (15)	0.44151 (14)	0.91443 (6)	0.0387 (4)	
C3	0.61548 (19)	0.40477 (17)	0.95811 (8)	0.0409 (4)	
C4	0.6128 (3)	0.3573 (2)	1.01395 (10)	0.0704 (8)	
H4A	0.6403	0.2820	1.0128	0.106*	
H4B	0.5319	0.3589	1.0263	0.106*	
H4C	0.6644	0.3989	1.0405	0.106*	
N4	0.3375 (2)	0.6038 (3)	0.56403 (10)	0.0857 (9)	
C5	0.2523 (3)	0.5621 (2)	0.57241 (10)	0.0576 (6)	
C6	0.1421 (3)	0.5092 (3)	0.58145 (13)	0.0799 (10)	
H6A	0.0807	0.5639	0.5858	0.120*	
H6B	0.1505	0.4647	0.6156	0.120*	
H6C	0.1200	0.4628	0.5490	0.120*	
N5	−0.1359 (7)	0.5306 (5)	1.0399 (3)	0.087 (3)	0.62
C7	−0.0520 (6)	0.5785 (5)	1.04825 (19)	0.0699 (16)	0.62
C8	0.0562 (13)	0.6419 (14)	1.0603 (7)	0.082 (4)	0.62
H8A	0.0494	0.6828	1.0954	0.123*	0.62
H8B	0.0672	0.6925	1.0292	0.123*	0.62
H8C	0.1239	0.5928	1.0643	0.123*	0.62
N5'	−0.1549 (9)	0.4866 (8)	1.0254 (4)	0.063 (2)	0.38
C7'	−0.1247 (6)	0.4336 (6)	0.9894 (3)	0.0539 (16)	0.38
C8'	−0.0796 (19)	0.3668 (18)	0.9442 (9)	0.061 (4)	0.38
H8D	−0.0008	0.3922	0.9356	0.091*	0.38
H8E	−0.1328	0.3726	0.9104	0.091*	0.38
H8F	−0.0749	0.2909	0.9564	0.091*	0.38
N11	0.80040 (13)	0.56775 (12)	0.85214 (6)	0.0302 (3)	
C11	0.89844 (17)	0.54836 (16)	0.82515 (8)	0.0339 (4)	
H11	0.8952	0.4946	0.7964	0.041*	
C12	1.00400 (17)	0.60162 (16)	0.83647 (7)	0.0340 (4)	
H12	1.0706	0.5846	0.8157	0.041*	
C13	1.01251 (16)	0.68036 (15)	0.87846 (7)	0.0293 (3)	
C14	0.91243 (17)	0.69721 (16)	0.90835 (7)	0.0333 (4)	
H14	0.9146	0.7476	0.9387	0.040*	
C15	0.80988 (17)	0.64104 (16)	0.89414 (7)	0.0332 (4)	
H15	0.7426	0.6548	0.9150	0.040*	
C16	1.12262 (16)	0.74442 (15)	0.89216 (7)	0.0324 (4)	
N12	1.21164 (14)	0.72915 (15)	0.85928 (7)	0.0397 (4)	
H1N	1.2027	0.6957	0.8266	0.060*	
H2N	1.2754	0.7687	0.8661	0.060*	
S11	1.13309 (5)	0.83113 (5)	0.94560 (2)	0.04363 (13)	
N21	0.45275 (13)	0.43047 (12)	0.80859 (6)	0.0304 (3)	
C21	0.37190 (16)	0.43714 (17)	0.84752 (8)	0.0365 (4)	
H21	0.3943	0.4691	0.8829	0.044*	
C22	0.25792 (17)	0.39979 (17)	0.83859 (8)	0.0378 (4)	
H22	0.2043	0.4034	0.8678	0.045*	
C23	0.22269 (16)	0.35688 (15)	0.78638 (8)	0.0329 (4)	
C24	0.30512 (17)	0.35158 (16)	0.74560 (8)	0.0341 (4)	
H24	0.2837	0.3240	0.7091	0.041*	
C25	0.41881 (16)	0.38689 (15)	0.75876 (7)	0.0317 (4)	
H25	0.4755	0.3799	0.7311	0.038*	
C26	0.09837 (16)	0.32242 (16)	0.77362 (8)	0.0362 (4)	
N22	0.05589 (16)	0.25230 (17)	0.80962 (9)	0.0536 (5)	
H3N	−0.0138	0.2228	0.8021	0.080*	
H4N	0.0964	0.2277	0.8396	0.080*	
S21	0.02293 (4)	0.37470 (4)	0.71886 (2)	0.03954 (12)	
N31	0.63857 (13)	0.54138 (12)	0.73865 (6)	0.0284 (3)	
C31	0.56230 (16)	0.61090 (15)	0.71318 (7)	0.0304 (4)	
H31	0.5006	0.6392	0.7342	0.037*	
C32	0.56864 (17)	0.64377 (15)	0.65764 (7)	0.0329 (4)	
H32	0.5107	0.6910	0.6407	0.039*	
C33	0.66128 (17)	0.60637 (15)	0.62729 (7)	0.0325 (4)	
C34	0.73991 (17)	0.53387 (16)	0.65314 (7)	0.0334 (4)	
H34	0.8040	0.5065	0.6334	0.040*	
C35	0.72442 (16)	0.50179 (15)	0.70767 (7)	0.0324 (4)	
H35	0.7768	0.4492	0.7243	0.039*	
C36	0.67792 (19)	0.64037 (17)	0.56790 (8)	0.0399 (4)	
N32	0.58298 (19)	0.63969 (19)	0.53393 (7)	0.0565 (5)	
H5N	0.5843	0.6602	0.4985	0.085*	
H6N	0.5141	0.6151	0.5435	0.085*	
S31	0.81118 (6)	0.67528 (6)	0.54887 (2)	0.05599 (16)	

### (Acetonitrile-κN)tris(pyridine-4-thioamide-κN)bis(thiocyanato-κN)cobalt(II) acetonitrile disolvate (Compound1) Atomic displacement parameters (Å^2^)

**Table:**

	*U*^11^	*U*^22^	*U*^33^	*U*^12^	*U*^13^	*U*^23^
Co1	0.02609 (12)	0.02759 (12)	0.02615 (11)	−0.00033 (9)	0.00155 (8)	−0.00024 (9)
N1	0.0378 (9)	0.0323 (8)	0.0376 (8)	0.0035 (7)	0.0024 (6)	0.0008 (6)
C1	0.0272 (8)	0.0343 (10)	0.0306 (8)	−0.0027 (7)	0.0019 (6)	0.0036 (7)
S1	0.0420 (3)	0.0280 (2)	0.0453 (2)	0.0007 (2)	0.0089 (2)	−0.00051 (19)
N2	0.0336 (8)	0.0337 (8)	0.0356 (7)	0.0017 (7)	0.0027 (6)	−0.0023 (6)
C2	0.0281 (9)	0.0343 (10)	0.0260 (7)	−0.0013 (7)	0.0008 (6)	0.0017 (7)
S2	0.0581 (3)	0.0324 (2)	0.0357 (2)	0.0065 (2)	0.0041 (2)	−0.00340 (19)
N3	0.0387 (9)	0.0450 (10)	0.0323 (8)	−0.0007 (8)	0.0018 (6)	0.0051 (7)
C3	0.0459 (11)	0.0408 (11)	0.0365 (10)	0.0055 (9)	0.0063 (8)	0.0020 (8)
C4	0.106 (2)	0.0678 (17)	0.0391 (11)	0.0193 (16)	0.0174 (13)	0.0178 (12)
N4	0.0587 (15)	0.141 (3)	0.0563 (13)	−0.0153 (16)	−0.0072 (11)	0.0269 (15)
C5	0.0606 (16)	0.0687 (17)	0.0430 (12)	−0.0035 (13)	−0.0019 (11)	0.0062 (11)
C6	0.099 (2)	0.081 (2)	0.0614 (16)	−0.0387 (19)	0.0166 (16)	−0.0124 (14)
N5	0.129 (7)	0.067 (4)	0.072 (4)	0.025 (4)	0.055 (4)	0.004 (3)
C7	0.101 (4)	0.062 (3)	0.051 (2)	0.038 (3)	0.041 (3)	0.021 (2)
C8	0.103 (9)	0.084 (6)	0.061 (4)	0.041 (6)	0.026 (5)	0.020 (4)
N5'	0.061 (4)	0.070 (7)	0.059 (5)	0.013 (4)	0.006 (4)	0.004 (4)
C7'	0.044 (3)	0.061 (4)	0.054 (4)	−0.002 (3)	−0.010 (3)	0.026 (3)
C8'	0.061 (7)	0.064 (8)	0.057 (9)	0.006 (7)	−0.003 (6)	0.016 (5)
N11	0.0289 (7)	0.0315 (8)	0.0300 (7)	−0.0015 (6)	0.0009 (6)	−0.0007 (6)
C11	0.0320 (9)	0.0357 (10)	0.0342 (9)	−0.0019 (8)	0.0031 (7)	−0.0070 (7)
C12	0.0300 (9)	0.0388 (10)	0.0335 (9)	−0.0020 (8)	0.0047 (7)	−0.0044 (7)
C13	0.0301 (9)	0.0312 (9)	0.0264 (8)	−0.0012 (7)	−0.0012 (6)	0.0033 (6)
C14	0.0342 (9)	0.0360 (10)	0.0296 (8)	−0.0003 (8)	0.0004 (7)	−0.0056 (7)
C15	0.0302 (9)	0.0392 (10)	0.0304 (8)	−0.0014 (8)	0.0035 (7)	−0.0039 (7)
C16	0.0333 (9)	0.0330 (9)	0.0304 (8)	−0.0019 (8)	−0.0027 (7)	0.0054 (7)
N12	0.0305 (8)	0.0489 (10)	0.0397 (8)	−0.0079 (7)	0.0009 (6)	−0.0052 (7)
S11	0.0460 (3)	0.0464 (3)	0.0382 (2)	−0.0120 (2)	0.0000 (2)	−0.0079 (2)
N21	0.0278 (7)	0.0309 (8)	0.0325 (7)	−0.0026 (6)	0.0015 (6)	−0.0017 (6)
C21	0.0308 (9)	0.0469 (11)	0.0319 (9)	−0.0028 (8)	0.0026 (7)	−0.0053 (8)
C22	0.0297 (9)	0.0481 (11)	0.0359 (9)	−0.0017 (8)	0.0046 (7)	−0.0020 (8)
C23	0.0292 (9)	0.0297 (9)	0.0395 (9)	−0.0006 (7)	−0.0004 (7)	0.0018 (7)
C24	0.0325 (9)	0.0348 (10)	0.0349 (9)	−0.0010 (8)	0.0008 (7)	−0.0032 (7)
C25	0.0307 (9)	0.0331 (9)	0.0315 (8)	−0.0023 (7)	0.0029 (7)	−0.0026 (7)
C26	0.0289 (9)	0.0351 (10)	0.0446 (10)	0.0002 (8)	0.0019 (7)	−0.0040 (8)
N22	0.0319 (9)	0.0620 (13)	0.0661 (12)	−0.0109 (9)	−0.0058 (8)	0.0201 (10)
S21	0.0325 (2)	0.0461 (3)	0.0393 (2)	0.0015 (2)	−0.00403 (18)	−0.0051 (2)
N31	0.0294 (7)	0.0308 (7)	0.0253 (6)	−0.0011 (6)	0.0026 (5)	0.0001 (5)
C31	0.0299 (9)	0.0317 (9)	0.0298 (8)	0.0007 (7)	0.0027 (7)	0.0004 (7)
C32	0.0344 (9)	0.0327 (9)	0.0313 (8)	0.0004 (8)	−0.0005 (7)	0.0017 (7)
C33	0.0373 (10)	0.0324 (9)	0.0279 (8)	−0.0075 (8)	0.0011 (7)	−0.0014 (7)
C34	0.0327 (9)	0.0382 (10)	0.0299 (8)	−0.0014 (8)	0.0051 (7)	−0.0032 (7)
C35	0.0305 (9)	0.0363 (10)	0.0304 (8)	0.0017 (8)	0.0024 (7)	−0.0001 (7)
C36	0.0496 (12)	0.0416 (11)	0.0288 (8)	−0.0045 (9)	0.0054 (8)	0.0012 (8)
N32	0.0536 (12)	0.0857 (15)	0.0298 (8)	−0.0040 (11)	−0.0003 (8)	0.0136 (9)
S31	0.0562 (3)	0.0740 (4)	0.0391 (3)	−0.0196 (3)	0.0143 (2)	0.0030 (3)

### (Acetonitrile-κN)tris(pyridine-4-thioamide-κN)bis(thiocyanato-κN)cobalt(II) acetonitrile disolvate (Compound1) Geometric parameters (Å, º)

**Table:**

Co1—N1	2.0650 (16)	C14—C15	1.380 (3)
Co1—N2	2.0720 (16)	C14—H14	0.9500
Co1—N21	2.1666 (15)	C15—H15	0.9500
Co1—N31	2.1785 (14)	C16—N12	1.326 (2)
Co1—N3	2.1950 (15)	C16—S11	1.6583 (19)
Co1—N11	2.2032 (15)	N12—H1N	0.8799
N1—C1	1.156 (2)	N12—H2N	0.8800
C1—S1	1.6378 (19)	N21—C25	1.336 (2)
N2—C2	1.155 (2)	N21—C21	1.342 (2)
C2—S2	1.6314 (19)	C21—C22	1.379 (3)
N3—C3	1.134 (2)	C21—H21	0.9500
C3—C4	1.451 (3)	C22—C23	1.386 (3)
C4—H4A	0.9800	C22—H22	0.9500
C4—H4B	0.9800	C23—C24	1.387 (3)
C4—H4C	0.9800	C23—C26	1.489 (3)
N4—C5	1.123 (4)	C24—C25	1.381 (3)
C5—C6	1.439 (4)	C24—H24	0.9500
C6—H6A	0.9800	C25—H25	0.9500
C6—H6B	0.9800	C26—N22	1.326 (3)
C6—H6C	0.9800	C26—S21	1.647 (2)
N5—C7	1.129 (8)	N22—H3N	0.8799
C7—C8	1.470 (14)	N22—H4N	0.8800
C8—H8A	0.9800	N31—C31	1.338 (2)
C8—H8B	0.9800	N31—C35	1.346 (2)
C8—H8C	0.9800	C31—C32	1.386 (2)
N5'—C7'	1.144 (9)	C31—H31	0.9500
C7'—C8'	1.469 (15)	C32—C33	1.388 (3)
C8'—H8D	0.9800	C32—H32	0.9500
C8'—H8E	0.9800	C33—C34	1.382 (3)
C8'—H8F	0.9800	C33—C36	1.494 (2)
N11—C11	1.338 (2)	C34—C35	1.375 (2)
N11—C15	1.346 (2)	C34—H34	0.9500
C11—C12	1.380 (3)	C35—H35	0.9500
C11—H11	0.9500	C36—N32	1.309 (3)
C12—C13	1.391 (3)	C36—S31	1.661 (2)
C12—H12	0.9500	N32—H5N	0.8800
C13—C14	1.390 (2)	N32—H6N	0.8799
C13—C16	1.499 (3)		
			
N1—Co1—N2	177.65 (6)	C15—C14—C13	120.22 (16)
N1—Co1—N21	91.08 (6)	C15—C14—H14	119.9
N2—Co1—N21	88.02 (6)	C13—C14—H14	119.9
N1—Co1—N31	89.52 (6)	N11—C15—C14	123.29 (16)
N2—Co1—N31	92.66 (6)	N11—C15—H15	118.4
N21—Co1—N31	90.26 (6)	C14—C15—H15	118.4
N1—Co1—N3	88.41 (6)	N12—C16—C13	116.87 (16)
N2—Co1—N3	89.39 (6)	N12—C16—S11	121.24 (15)
N21—Co1—N3	88.82 (6)	C13—C16—S11	121.88 (13)
N31—Co1—N3	177.72 (6)	C16—N12—H1N	122.2
N1—Co1—N11	92.70 (6)	C16—N12—H2N	117.4
N2—Co1—N11	88.06 (6)	H1N—N12—H2N	118.3
N21—Co1—N11	174.69 (5)	C25—N21—C21	117.44 (16)
N31—Co1—N11	93.50 (5)	C25—N21—Co1	122.63 (12)
N3—Co1—N11	87.56 (6)	C21—N21—Co1	119.88 (12)
C1—N1—Co1	173.25 (16)	N21—C21—C22	123.13 (17)
N1—C1—S1	179.26 (19)	N21—C21—H21	118.4
C2—N2—Co1	166.49 (16)	C22—C21—H21	118.4
N2—C2—S2	179.66 (17)	C21—C22—C23	119.05 (17)
C3—N3—Co1	173.73 (17)	C21—C22—H22	120.5
N3—C3—C4	179.7 (3)	C23—C22—H22	120.5
C3—C4—H4A	109.5	C22—C23—C24	118.11 (17)
C3—C4—H4B	109.5	C22—C23—C26	120.86 (16)
H4A—C4—H4B	109.5	C24—C23—C26	120.96 (16)
C3—C4—H4C	109.5	C25—C24—C23	119.13 (17)
H4A—C4—H4C	109.5	C25—C24—H24	120.4
H4B—C4—H4C	109.5	C23—C24—H24	120.4
N4—C5—C6	178.3 (3)	N21—C25—C24	123.06 (16)
C5—C6—H6A	109.5	N21—C25—H25	118.5
C5—C6—H6B	109.5	C24—C25—H25	118.5
H6A—C6—H6B	109.5	N22—C26—C23	115.48 (17)
C5—C6—H6C	109.5	N22—C26—S21	124.91 (16)
H6A—C6—H6C	109.5	C23—C26—S21	119.58 (14)
H6B—C6—H6C	109.5	C26—N22—H3N	119.6
N5—C7—C8	178.7 (8)	C26—N22—H4N	123.7
C7—C8—H8A	109.5	H3N—N22—H4N	116.4
C7—C8—H8B	109.5	C31—N31—C35	117.08 (14)
H8A—C8—H8B	109.5	C31—N31—Co1	122.23 (11)
C7—C8—H8C	109.5	C35—N31—Co1	120.68 (12)
H8A—C8—H8C	109.5	N31—C31—C32	123.38 (16)
H8B—C8—H8C	109.5	N31—C31—H31	118.3
N5'—C7'—C8'	177.0 (11)	C32—C31—H31	118.3
C7'—C8'—H8D	109.5	C31—C32—C33	118.66 (17)
C7'—C8'—H8E	109.5	C31—C32—H32	120.7
H8D—C8'—H8E	109.5	C33—C32—H32	120.7
C7'—C8'—H8F	109.5	C34—C33—C32	118.28 (16)
H8D—C8'—H8F	109.5	C34—C33—C36	119.17 (17)
H8E—C8'—H8F	109.5	C32—C33—C36	122.54 (18)
C11—N11—C15	116.21 (16)	C35—C34—C33	119.35 (17)
C11—N11—Co1	123.08 (12)	C35—C34—H34	120.3
C15—N11—Co1	120.56 (12)	C33—C34—H34	120.3
N11—C11—C12	124.01 (17)	N31—C35—C34	123.11 (17)
N11—C11—H11	118.0	N31—C35—H35	118.4
C12—C11—H11	118.0	C34—C35—H35	118.4
C11—C12—C13	119.72 (17)	N32—C36—C33	115.85 (18)
C11—C12—H12	120.1	N32—C36—S31	124.40 (15)
C13—C12—H12	120.1	C33—C36—S31	119.75 (15)
C14—C13—C12	116.44 (17)	C36—N32—H5N	121.9
C14—C13—C16	120.45 (16)	C36—N32—H6N	123.8
C12—C13—C16	123.11 (16)	H5N—N32—H6N	114.2

### (Acetonitrile-κN)tris(pyridine-4-thioamide-κN)bis(thiocyanato-κN)cobalt(II) acetonitrile disolvate (Compound1) Hydrogen-bond geometry (Å, º)

**Table:**

*D*—H···*A*	*D*—H	H···*A*	*D*···*A*	*D*—H···*A*
C4—H4*C*···N5′^i^	0.98	2.37	3.081 (12)	129
C6—H6*C*···S31^ii^	0.98	3.02	3.901 (3)	150
C11—H11···S21^i^	0.95	2.83	3.6556 (18)	146
C12—H12···S1^iii^	0.95	3.01	3.8491 (18)	148
N12—H1*N*···S1^iii^	0.88	2.66	3.5097 (17)	163
N12—H2*N*···S2^i^	0.88	2.71	3.5731 (17)	167
C21—H21···N3	0.95	2.63	3.134 (3)	114
C22—H22···N5^iv^	0.95	2.50	3.384 (7)	154
C25—H25···S2^v^	0.95	2.91	3.7172 (18)	144
N22—H3*N*···S1^vi^	0.88	2.59	3.4715 (19)	179
N22—H4*N*···S31^v^	0.88	2.87	3.729 (2)	167
C34—H34···S11^vii^	0.95	2.98	3.7698 (19)	142
C35—H35···N1	0.95	2.56	3.094 (2)	116
C35—H35···S21^i^	0.95	2.95	3.7301 (19)	140
N32—H5*N*···S2^viii^	0.88	2.74	3.5390 (18)	152
N32—H6*N*···N4	0.88	2.10	2.951 (3)	164
C8—H8*B*···S11^vi^	0.98	2.76	3.728 (19)	172
C8′—H8*D*···N5′^iv^	0.98	2.46	3.26 (2)	140
C8′—H8*F*···S11^ix^	0.98	2.88	3.65 (3)	137

Symmetry codes: (i) *x*+1, *y*, *z*; (ii) −*x*+1, −*y*+1, −*z*+1; (iii) −*x*+2, *y*+1/2, −*z*+3/2; (iv) −*x*, −*y*+1, −*z*+2; (v) −*x*+1, *y*−1/2, −*z*+3/2; (vi) *x*−1, *y*, *z*; (vii) −*x*+2, *y*−1/2, −*z*+3/2; (viii) *x*, −*y*+3/2, *z*−1/2; (ix) −*x*+1, −*y*+1, −*z*+2.

### Tetrakis(pyridine-4-thioamide-κN)bis(thiocyanato-κN)nickel(II) methanol pentasolvate (Compound2) Crystal data

**Table:**

[Ni(NCS)_2_(C_6_H_6_N_2_S)_4_]·5CH_4_O	*Z* = 2
*M**_r_* = 887.83	*F*(000) = 928
Triclinic, *P*1	*D*_x_ = 1.392 Mg m^−^^3^
*a* = 10.4520 (3) Å	Mo *K*α radiation, λ = 0.71073 Å
*b* = 14.5934 (4) Å	Cell parameters from 30865 reflections
*c* = 15.0580 (5) Å	θ = 1.5–27.0°
α = 101.553 (2)°	µ = 0.80 mm^−^^1^
β = 97.105 (2)°	*T* = 200 K
γ = 106.417 (2)°	Block, yellow
*V* = 2118.43 (11) Å^3^	0.30 × 0.18 × 0.10 mm

### Tetrakis(pyridine-4-thioamide-κN)bis(thiocyanato-κN)nickel(II) methanol pentasolvate (Compound2) Data collection

**Table:**

STOE IPDS-2 diffractometer	7895 reflections with *I* > 2σ(*I*)
ω scans	*R*_int_ = 0.031
Absorption correction: numerical (X-Red and X-Shape; Stoe, 2008)	θ_max_ = 27.0°, θ_min_ = 1.5°
*T*_min_ = 0.622, *T*_max_ = 0.889	*h* = −13→13
30865 measured reflections	*k* = −18→18
9253 independent reflections	*l* = −19→19

### Tetrakis(pyridine-4-thioamide-κN)bis(thiocyanato-κN)nickel(II) methanol pentasolvate (Compound2) Refinement

**Table:**

Refinement on *F*^2^	0 restraints
Least-squares matrix: full	Hydrogen site location: mixed
*R*[*F*^2^ > 2σ(*F*^2^)] = 0.039	H-atom parameters constrained
*wR*(*F*^2^) = 0.109	*w* = 1/[σ^2^(*F*_o_^2^) + (0.0575*P*)^2^ + 1.0537*P*] where *P* = (*F*_o_^2^ + 2*F*_c_^2^)/3
*S* = 1.06	(Δ/σ)_max_ = 0.026
9253 reflections	Δρ_max_ = 0.62 e Å^−^^3^
486 parameters	Δρ_min_ = −0.57 e Å^−^^3^

### Tetrakis(pyridine-4-thioamide-κN)bis(thiocyanato-κN)nickel(II) methanol pentasolvate (Compound2) Special details

**Table:**

Geometry. All esds (except the esd in the dihedral angle between two l.s. planes) are estimated using the full covariance matrix. The cell esds are taken into account individually in the estimation of esds in distances, angles and torsion angles; correlations between esds in cell parameters are only used when they are defined by crystal symmetry. An approximate (isotropic) treatment of cell esds is used for estimating esds involving l.s. planes.

### Tetrakis(pyridine-4-thioamide-κN)bis(thiocyanato-κN)nickel(II) methanol pentasolvate (Compound2) Fractional atomic coordinates and isotropic or equivalent isotropic displacement parameters (Å^2^)

**Table:**

	*x*	*y*	*z*	*U*_iso_*/*U*_eq_	
Ni1	0.51341 (2)	0.75649 (2)	0.25835 (2)	0.02541 (8)	
N1	0.54979 (18)	0.69614 (13)	0.13314 (13)	0.0327 (4)	
C1	0.55335 (19)	0.66218 (14)	0.05750 (14)	0.0274 (4)	
S1	0.55930 (7)	0.61468 (4)	−0.04898 (4)	0.03984 (14)	
N2	0.47316 (17)	0.81774 (12)	0.38258 (12)	0.0312 (4)	
C2	0.45784 (19)	0.84815 (14)	0.45634 (14)	0.0270 (4)	
S2	0.43803 (5)	0.89172 (4)	0.56086 (4)	0.03231 (12)	
O1	1.11056 (19)	0.76700 (17)	0.45151 (19)	0.0685 (6)	
H1	1.1714	0.7529	0.4827	0.103*	
C3	0.9826 (3)	0.7028 (2)	0.4528 (3)	0.0619 (8)	
H3A	0.9932	0.6612	0.4951	0.093*	
H3B	0.9412	0.6610	0.3905	0.093*	
H3C	0.9240	0.7413	0.4737	0.093*	
O2	0.2866 (3)	0.29719 (16)	0.25572 (19)	0.0746 (7)	
H2	0.2560	0.2466	0.2117	0.112*	
C4	0.2076 (4)	0.3592 (3)	0.2485 (3)	0.0869 (11)	
H4A	0.2268	0.4087	0.3071	0.130*	
H4B	0.1111	0.3202	0.2341	0.130*	
H4C	0.2294	0.3923	0.1991	0.130*	
O3	−0.1063 (2)	0.74182 (19)	0.0459 (2)	0.0769 (7)	
H3	−0.1766	0.7415	0.0120	0.115*	
C5	0.0077 (4)	0.7993 (3)	0.0215 (4)	0.0954 (14)	
H5A	−0.0007	0.8647	0.0224	0.143*	
H5B	0.0151	0.7679	−0.0407	0.143*	
H5C	0.0891	0.8060	0.0655	0.143*	
O4	0.9917 (4)	1.1226 (3)	0.1994 (3)	0.1172 (11)	
H4	0.9255	1.1374	0.2174	0.176*	
C6	0.9483 (5)	1.0242 (3)	0.1498 (3)	0.0990 (14)	
H6A	1.0269	1.0004	0.1471	0.149*	
H6B	0.9032	1.0186	0.0869	0.149*	
H6C	0.8843	0.9844	0.1804	0.149*	
O5	0.7792 (3)	1.1926 (2)	0.2133 (2)	0.0917 (9)	
H5	0.8183	1.2328	0.1847	0.138*	
C7	0.8085 (7)	1.2391 (5)	0.3032 (4)	0.138 (2)	
H7A	0.8012	1.3054	0.3079	0.208*	
H7B	0.9017	1.2459	0.3307	0.208*	
H7C	0.7455	1.2058	0.3385	0.208*	
N11	0.55562 (17)	0.64292 (12)	0.31467 (12)	0.0292 (3)	
C11	0.5353 (3)	0.55466 (16)	0.25963 (16)	0.0399 (5)	
H11	0.5032	0.5446	0.1955	0.048*	
C12	0.5581 (3)	0.47683 (17)	0.29056 (16)	0.0428 (5)	
H12	0.5421	0.4151	0.2483	0.051*	
C13	0.6048 (2)	0.48958 (16)	0.38378 (15)	0.0336 (4)	
C14	0.6301 (2)	0.58205 (16)	0.44148 (15)	0.0355 (5)	
H14	0.6653	0.5947	0.5055	0.043*	
C15	0.6037 (2)	0.65570 (16)	0.40479 (15)	0.0340 (4)	
H15	0.6202	0.7185	0.4452	0.041*	
C16	0.6263 (3)	0.40563 (17)	0.41981 (16)	0.0398 (5)	
N12	0.5319 (2)	0.31979 (15)	0.38285 (15)	0.0457 (5)	
H1N	0.4568	0.3147	0.3458	0.069*	
H2N	0.5292	0.2622	0.3934	0.069*	
S11	0.76130 (8)	0.42440 (6)	0.50051 (6)	0.0593 (2)	
N21	0.72138 (16)	0.84055 (12)	0.30150 (12)	0.0286 (3)	
C21	0.8184 (2)	0.80324 (16)	0.27672 (16)	0.0342 (4)	
H21	0.7920	0.7383	0.2383	0.041*	
C22	0.9556 (2)	0.85515 (16)	0.30453 (16)	0.0363 (5)	
H22	1.0218	0.8256	0.2868	0.044*	
C23	0.9951 (2)	0.95123 (16)	0.35878 (15)	0.0315 (4)	
C24	0.8945 (2)	0.99054 (15)	0.38322 (15)	0.0329 (4)	
H24	0.9178	1.0564	0.4192	0.040*	
C25	0.7602 (2)	0.93304 (15)	0.35472 (15)	0.0315 (4)	
H25	0.6922	0.9599	0.3735	0.038*	
C26	1.1412 (2)	1.01136 (16)	0.38800 (15)	0.0344 (4)	
N22	1.22398 (18)	0.96366 (15)	0.41365 (14)	0.0385 (4)	
H4N	1.3129	0.9904	0.4262	0.058*	
H3N	1.1961	0.9051	0.4250	0.058*	
S21	1.19199 (6)	1.12876 (4)	0.38386 (5)	0.04608 (15)	
N31	0.47817 (17)	0.87293 (12)	0.20441 (12)	0.0288 (3)	
C31	0.5552 (2)	0.91431 (15)	0.14905 (15)	0.0324 (4)	
H31	0.6224	0.8868	0.1296	0.039*	
C32	0.5413 (2)	0.99481 (16)	0.11913 (16)	0.0359 (5)	
H32	0.5997	1.0230	0.0814	0.043*	
C33	0.4415 (2)	1.03440 (16)	0.14444 (16)	0.0345 (4)	
C34	0.3592 (2)	0.99038 (17)	0.20028 (16)	0.0374 (5)	
H34	0.2884	1.0144	0.2183	0.045*	
C35	0.3820 (2)	0.91149 (16)	0.22892 (15)	0.0339 (4)	
H35	0.3266	0.8830	0.2681	0.041*	
C36	0.4190 (2)	1.11957 (17)	0.11202 (17)	0.0403 (5)	
N32	0.5284 (2)	1.19414 (15)	0.11852 (18)	0.0520 (6)	
H5N	0.6061	1.1934	0.1481	0.078*	
H6N	0.5270	1.2440	0.0943	0.078*	
S31	0.26248 (7)	1.11340 (5)	0.06808 (6)	0.0578 (2)	
N41	0.30423 (17)	0.67290 (13)	0.21366 (12)	0.0310 (4)	
C41	0.2366 (2)	0.66188 (16)	0.12907 (15)	0.0342 (4)	
H41	0.2852	0.6901	0.0866	0.041*	
C42	0.0993 (2)	0.61148 (16)	0.10008 (16)	0.0370 (5)	
H42	0.0543	0.6076	0.0400	0.044*	
C43	0.0283 (2)	0.56666 (16)	0.15998 (17)	0.0361 (5)	
C44	0.0988 (2)	0.57619 (17)	0.24755 (17)	0.0398 (5)	
H44	0.0540	0.5457	0.2902	0.048*	
C45	0.2346 (2)	0.63047 (16)	0.27168 (16)	0.0363 (5)	
H45	0.2812	0.6382	0.3324	0.044*	
C46	−0.1192 (2)	0.50686 (18)	0.13038 (19)	0.0437 (5)	
N42	−0.1973 (2)	0.54523 (17)	0.08483 (17)	0.0515 (5)	
H7N	−0.1699	0.6010	0.0683	0.077*	
H8N	−0.2852	0.5139	0.0721	0.077*	
S41	−0.17085 (7)	0.39687 (6)	0.15227 (7)	0.0666 (2)	

### Tetrakis(pyridine-4-thioamide-κN)bis(thiocyanato-κN)nickel(II) methanol pentasolvate (Compound2) Atomic displacement parameters (Å^2^)

**Table:**

	*U*^11^	*U*^22^	*U*^33^	*U*^12^	*U*^13^	*U*^23^
Ni1	0.02585 (13)	0.02370 (13)	0.02562 (13)	0.00738 (9)	0.00263 (9)	0.00581 (9)
N1	0.0353 (9)	0.0315 (9)	0.0308 (9)	0.0112 (7)	0.0047 (7)	0.0065 (7)
C1	0.0274 (9)	0.0241 (9)	0.0301 (10)	0.0079 (7)	0.0012 (7)	0.0087 (8)
S1	0.0564 (3)	0.0380 (3)	0.0263 (3)	0.0188 (3)	0.0059 (2)	0.0062 (2)
N2	0.0327 (9)	0.0288 (8)	0.0318 (9)	0.0103 (7)	0.0055 (7)	0.0063 (7)
C2	0.0243 (8)	0.0228 (9)	0.0337 (11)	0.0073 (7)	0.0023 (7)	0.0089 (8)
S2	0.0369 (3)	0.0324 (3)	0.0302 (3)	0.0145 (2)	0.0078 (2)	0.0076 (2)
O1	0.0363 (9)	0.0662 (13)	0.1032 (18)	0.0093 (9)	−0.0012 (10)	0.0403 (13)
C3	0.0440 (14)	0.0586 (17)	0.082 (2)	0.0114 (13)	0.0091 (14)	0.0225 (16)
O2	0.0758 (15)	0.0506 (12)	0.0884 (18)	0.0191 (11)	−0.0121 (12)	0.0152 (11)
C4	0.072 (2)	0.080 (3)	0.110 (3)	0.029 (2)	0.010 (2)	0.021 (2)
O3	0.0504 (11)	0.0750 (15)	0.109 (2)	0.0153 (11)	−0.0006 (12)	0.0485 (15)
C5	0.062 (2)	0.088 (3)	0.153 (4)	0.0253 (19)	0.014 (2)	0.070 (3)
O4	0.109 (2)	0.115 (3)	0.120 (3)	0.049 (2)	−0.005 (2)	0.008 (2)
C6	0.087 (3)	0.101 (3)	0.083 (3)	0.001 (2)	0.006 (2)	0.014 (2)
O5	0.0716 (16)	0.0709 (16)	0.119 (2)	0.0153 (13)	−0.0155 (15)	0.0239 (16)
C7	0.186 (6)	0.197 (6)	0.094 (4)	0.101 (5)	0.082 (4)	0.079 (4)
N11	0.0326 (8)	0.0260 (8)	0.0280 (8)	0.0093 (7)	0.0016 (7)	0.0069 (6)
C11	0.0605 (14)	0.0289 (10)	0.0282 (11)	0.0141 (10)	0.0019 (10)	0.0063 (8)
C12	0.0683 (16)	0.0283 (10)	0.0330 (11)	0.0187 (10)	0.0061 (11)	0.0076 (9)
C13	0.0369 (10)	0.0334 (10)	0.0347 (11)	0.0141 (8)	0.0067 (9)	0.0134 (9)
C14	0.0408 (11)	0.0382 (11)	0.0278 (10)	0.0151 (9)	0.0003 (8)	0.0087 (8)
C15	0.0387 (11)	0.0307 (10)	0.0303 (10)	0.0126 (8)	−0.0010 (8)	0.0046 (8)
C16	0.0509 (13)	0.0387 (12)	0.0380 (12)	0.0216 (10)	0.0107 (10)	0.0160 (9)
N12	0.0586 (13)	0.0343 (10)	0.0495 (12)	0.0189 (9)	0.0064 (10)	0.0182 (9)
S11	0.0673 (4)	0.0537 (4)	0.0597 (4)	0.0251 (3)	−0.0083 (3)	0.0239 (3)
N21	0.0260 (8)	0.0273 (8)	0.0307 (9)	0.0077 (6)	0.0030 (6)	0.0053 (7)
C21	0.0310 (10)	0.0297 (10)	0.0386 (11)	0.0114 (8)	0.0024 (8)	0.0009 (8)
C22	0.0291 (10)	0.0355 (11)	0.0428 (12)	0.0138 (8)	0.0054 (9)	0.0026 (9)
C23	0.0276 (9)	0.0341 (10)	0.0306 (10)	0.0084 (8)	0.0034 (8)	0.0063 (8)
C24	0.0305 (10)	0.0293 (10)	0.0354 (11)	0.0080 (8)	0.0053 (8)	0.0029 (8)
C25	0.0281 (9)	0.0289 (10)	0.0363 (11)	0.0098 (8)	0.0057 (8)	0.0042 (8)
C26	0.0286 (10)	0.0387 (11)	0.0317 (11)	0.0087 (8)	0.0053 (8)	0.0023 (9)
N22	0.0256 (8)	0.0417 (10)	0.0452 (11)	0.0101 (7)	0.0023 (7)	0.0076 (8)
S21	0.0317 (3)	0.0354 (3)	0.0643 (4)	0.0052 (2)	0.0027 (3)	0.0088 (3)
N31	0.0300 (8)	0.0277 (8)	0.0301 (9)	0.0096 (7)	0.0044 (7)	0.0106 (7)
C31	0.0306 (10)	0.0330 (10)	0.0375 (11)	0.0123 (8)	0.0091 (8)	0.0123 (9)
C32	0.0358 (10)	0.0336 (11)	0.0411 (12)	0.0100 (8)	0.0091 (9)	0.0157 (9)
C33	0.0357 (10)	0.0299 (10)	0.0372 (11)	0.0105 (8)	0.0011 (9)	0.0101 (8)
C34	0.0391 (11)	0.0384 (11)	0.0412 (12)	0.0199 (9)	0.0099 (9)	0.0116 (9)
C35	0.0352 (10)	0.0368 (11)	0.0354 (11)	0.0159 (9)	0.0104 (9)	0.0127 (9)
C36	0.0462 (12)	0.0348 (11)	0.0432 (13)	0.0171 (10)	0.0038 (10)	0.0139 (10)
N32	0.0485 (12)	0.0370 (11)	0.0726 (16)	0.0118 (9)	0.0008 (11)	0.0269 (10)
S31	0.0447 (3)	0.0499 (4)	0.0843 (5)	0.0191 (3)	−0.0010 (3)	0.0312 (4)
N41	0.0283 (8)	0.0304 (8)	0.0317 (9)	0.0061 (7)	0.0020 (7)	0.0088 (7)
C41	0.0316 (10)	0.0360 (11)	0.0325 (11)	0.0070 (8)	0.0020 (8)	0.0106 (9)
C42	0.0326 (10)	0.0377 (11)	0.0368 (11)	0.0081 (9)	−0.0022 (9)	0.0105 (9)
C43	0.0287 (10)	0.0315 (10)	0.0452 (13)	0.0075 (8)	0.0027 (9)	0.0087 (9)
C44	0.0349 (11)	0.0414 (12)	0.0410 (12)	0.0057 (9)	0.0070 (9)	0.0150 (10)
C45	0.0338 (10)	0.0383 (11)	0.0333 (11)	0.0056 (9)	0.0016 (8)	0.0129 (9)
C46	0.0311 (11)	0.0405 (12)	0.0548 (15)	0.0066 (9)	0.0026 (10)	0.0116 (11)
N42	0.0308 (10)	0.0495 (12)	0.0692 (15)	0.0080 (9)	−0.0023 (10)	0.0176 (11)
S41	0.0384 (3)	0.0507 (4)	0.1036 (7)	−0.0017 (3)	−0.0032 (4)	0.0362 (4)

### Tetrakis(pyridine-4-thioamide-κN)bis(thiocyanato-κN)nickel(II) methanol pentasolvate (Compound2) Geometric parameters (Å, º)

**Table:**

Ni1—N1	2.0435 (18)	N12—H1N	0.8800
Ni1—N2	2.0526 (18)	N12—H2N	0.8801
Ni1—N21	2.1157 (16)	N21—C21	1.337 (3)
Ni1—N31	2.1250 (17)	N21—C25	1.344 (3)
Ni1—N41	2.1262 (17)	C21—C22	1.384 (3)
Ni1—N11	2.1316 (17)	C21—H21	0.9500
N1—C1	1.157 (3)	C22—C23	1.391 (3)
C1—S1	1.631 (2)	C22—H22	0.9500
N2—C2	1.159 (3)	C23—C24	1.387 (3)
C2—S2	1.636 (2)	C23—C26	1.490 (3)
O1—C3	1.405 (3)	C24—C25	1.378 (3)
O1—H1	0.8400	C24—H24	0.9500
C3—H3A	0.9800	C25—H25	0.9500
C3—H3B	0.9800	C26—N22	1.322 (3)
C3—H3C	0.9800	C26—S21	1.661 (2)
O2—C4	1.398 (4)	N22—H4N	0.8800
O2—H2	0.8400	N22—H3N	0.8801
C4—H4A	0.9800	N31—C35	1.339 (3)
C4—H4B	0.9800	N31—C31	1.342 (3)
C4—H4C	0.9800	C31—C32	1.377 (3)
O3—C5	1.388 (4)	C31—H31	0.9500
O3—H3	0.8400	C32—C33	1.385 (3)
C5—H5A	0.9800	C32—H32	0.9500
C5—H5B	0.9800	C33—C34	1.394 (3)
C5—H5C	0.9800	C33—C36	1.489 (3)
O4—C6	1.395 (5)	C34—C35	1.377 (3)
O4—H4	0.8400	C34—H34	0.9500
C6—H6A	0.9800	C35—H35	0.9500
C6—H6B	0.9800	C36—N32	1.315 (3)
C6—H6C	0.9800	C36—S31	1.657 (2)
O5—C7	1.341 (6)	N32—H5N	0.8801
O5—H5	0.8401	N32—H6N	0.8799
C7—H7A	0.9800	N41—C41	1.334 (3)
C7—H7B	0.9800	N41—C45	1.340 (3)
C7—H7C	0.9800	C41—C42	1.383 (3)
N11—C11	1.329 (3)	C41—H41	0.9500
N11—C15	1.344 (3)	C42—C43	1.385 (3)
C11—C12	1.379 (3)	C42—H42	0.9500
C11—H11	0.9500	C43—C44	1.389 (3)
C12—C13	1.385 (3)	C43—C46	1.501 (3)
C12—H12	0.9500	C44—C45	1.377 (3)
C13—C14	1.385 (3)	C44—H44	0.9500
C13—C16	1.496 (3)	C45—H45	0.9500
C14—C15	1.380 (3)	C46—N42	1.313 (3)
C14—H14	0.9500	C46—S41	1.656 (3)
C15—H15	0.9500	N42—H7N	0.8799
C16—N12	1.319 (3)	N42—H8N	0.8800
C16—S11	1.663 (3)		
			
N1—Ni1—N2	178.69 (7)	C16—N12—H1N	121.7
N1—Ni1—N21	90.21 (7)	C16—N12—H2N	127.8
N2—Ni1—N21	90.67 (7)	H1N—N12—H2N	110.4
N1—Ni1—N31	89.06 (7)	C21—N21—C25	117.90 (17)
N2—Ni1—N31	89.98 (7)	C21—N21—Ni1	121.02 (13)
N21—Ni1—N31	89.16 (6)	C25—N21—Ni1	121.08 (13)
N1—Ni1—N41	89.46 (7)	N21—C21—C22	122.89 (19)
N2—Ni1—N41	89.65 (7)	N21—C21—H21	118.6
N21—Ni1—N41	179.30 (7)	C22—C21—H21	118.6
N31—Ni1—N41	90.22 (7)	C21—C22—C23	118.92 (19)
N1—Ni1—N11	91.28 (7)	C21—C22—H22	120.5
N2—Ni1—N11	89.71 (7)	C23—C22—H22	120.5
N21—Ni1—N11	88.91 (6)	C24—C23—C22	118.20 (19)
N31—Ni1—N11	178.04 (6)	C24—C23—C26	120.74 (19)
N41—Ni1—N11	91.72 (7)	C22—C23—C26	121.04 (19)
C1—N1—Ni1	170.85 (17)	C25—C24—C23	119.27 (19)
N1—C1—S1	179.7 (2)	C25—C24—H24	120.4
C2—N2—Ni1	173.81 (17)	C23—C24—H24	120.4
N2—C2—S2	179.4 (2)	N21—C25—C24	122.78 (19)
C3—O1—H1	109.5	N21—C25—H25	118.6
O1—C3—H3A	109.5	C24—C25—H25	118.6
O1—C3—H3B	109.5	N22—C26—C23	115.3 (2)
H3A—C3—H3B	109.5	N22—C26—S21	124.16 (17)
O1—C3—H3C	109.5	C23—C26—S21	120.56 (17)
H3A—C3—H3C	109.5	C26—N22—H4N	122.5
H3B—C3—H3C	109.5	C26—N22—H3N	123.7
C4—O2—H2	109.5	H4N—N22—H3N	113.5
O2—C4—H4A	109.5	C35—N31—C31	117.33 (18)
O2—C4—H4B	109.5	C35—N31—Ni1	120.65 (14)
H4A—C4—H4B	109.5	C31—N31—Ni1	121.92 (14)
O2—C4—H4C	109.5	N31—C31—C32	123.0 (2)
H4A—C4—H4C	109.5	N31—C31—H31	118.5
H4B—C4—H4C	109.5	C32—C31—H31	118.5
C5—O3—H3	109.5	C31—C32—C33	119.5 (2)
O3—C5—H5A	109.5	C31—C32—H32	120.2
O3—C5—H5B	109.5	C33—C32—H32	120.2
H5A—C5—H5B	109.5	C32—C33—C34	117.7 (2)
O3—C5—H5C	109.5	C32—C33—C36	122.0 (2)
H5A—C5—H5C	109.5	C34—C33—C36	120.3 (2)
H5B—C5—H5C	109.5	C35—C34—C33	119.0 (2)
C6—O4—H4	109.5	C35—C34—H34	120.5
O4—C6—H6A	109.5	C33—C34—H34	120.5
O4—C6—H6B	109.5	N31—C35—C34	123.4 (2)
H6A—C6—H6B	109.5	N31—C35—H35	118.3
O4—C6—H6C	109.5	C34—C35—H35	118.3
H6A—C6—H6C	109.5	N32—C36—C33	116.1 (2)
H6B—C6—H6C	109.5	N32—C36—S31	124.75 (19)
C7—O5—H5	107.6	C33—C36—S31	119.16 (17)
O5—C7—H7A	107.6	C36—N32—H5N	118.1
O5—C7—H7B	110.9	C36—N32—H6N	122.8
H7A—C7—H7B	108.2	H5N—N32—H6N	119.1
O5—C7—H7C	112.6	C41—N41—C45	117.46 (18)
H7A—C7—H7C	108.2	C41—N41—Ni1	122.39 (14)
H7B—C7—H7C	109.3	C45—N41—Ni1	120.14 (14)
C11—N11—C15	116.96 (18)	N41—C41—C42	123.2 (2)
C11—N11—Ni1	119.87 (14)	N41—C41—H41	118.4
C15—N11—Ni1	123.18 (14)	C42—C41—H41	118.4
N11—C11—C12	123.6 (2)	C41—C42—C43	119.0 (2)
N11—C11—H11	118.2	C41—C42—H42	120.5
C12—C11—H11	118.2	C43—C42—H42	120.5
C11—C12—C13	119.3 (2)	C42—C43—C44	118.0 (2)
C11—C12—H12	120.3	C42—C43—C46	121.1 (2)
C13—C12—H12	120.3	C44—C43—C46	120.8 (2)
C14—C13—C12	117.7 (2)	C45—C44—C43	119.1 (2)
C14—C13—C16	121.8 (2)	C45—C44—H44	120.4
C12—C13—C16	120.5 (2)	C43—C44—H44	120.4
C15—C14—C13	119.2 (2)	N41—C45—C44	123.1 (2)
C15—C14—H14	120.4	N41—C45—H45	118.4
C13—C14—H14	120.4	C44—C45—H45	118.4
N11—C15—C14	123.3 (2)	N42—C46—C43	116.3 (2)
N11—C15—H15	118.4	N42—C46—S41	124.76 (18)
C14—C15—H15	118.4	C43—C46—S41	118.88 (18)
N12—C16—C13	114.9 (2)	C46—N42—H7N	125.7
N12—C16—S11	124.92 (18)	C46—N42—H8N	117.1
C13—C16—S11	120.23 (18)	H7N—N42—H8N	117.1

### Tetrakis(pyridine-4-thioamide-κN)bis(thiocyanato-κN)nickel(II) methanol pentasolvate (Compound2) Hydrogen-bond geometry (Å, º)

**Table:**

*D*—H···*A*	*D*—H	H···*A*	*D*···*A*	*D*—H···*A*
O1—H1···S2^i^	0.84	2.88	3.409 (2)	123
O1—H1···S11^ii^	0.84	2.92	3.578 (2)	137
O2—H2···S31^iii^	0.84	2.62	3.413 (3)	157
O3—H3···S1^iv^	0.84	2.77	3.427 (2)	136
O3—H3···S31^v^	0.84	2.93	3.576 (2)	135
C5—H5*A*···S31^v^	0.98	3.03	3.632 (4)	121
O4—H4···O5	0.84	1.92	2.708 (5)	156
C6—H6*B*···S31^vi^	0.98	2.73	3.566 (4)	143
O5—H5···S41^vii^	0.84	2.51	3.216 (3)	142
C7—H7*A*···S41^vii^	0.98	2.93	3.528 (5)	121
C11—H11···N1	0.95	2.52	3.063 (3)	117
C11—H11···S1^viii^	0.95	2.73	3.442 (2)	133
C12—H12···S1^viii^	0.95	2.96	3.542 (2)	121
C15—H15···N2	0.95	2.61	3.097 (3)	113
N12—H1*N*···O2	0.88	2.02	2.898 (3)	177
N12—H2*N*···S2^ix^	0.88	2.58	3.446 (2)	171
C21—H21···N1	0.95	2.65	3.109 (3)	110
C25—H25···N2	0.95	2.66	3.122 (3)	111
C25—H25···S2^x^	0.95	2.94	3.846 (2)	159
N22—H4*N*···S2^xi^	0.88	2.64	3.4939 (19)	163
N22—H3*N*···O1	0.88	2.10	2.978 (3)	174
N32—H5*N*···O5	0.88	1.95	2.833 (3)	180
N32—H6*N*···S1^vi^	0.88	2.64	3.478 (2)	159
C45—H45···S11^ix^	0.95	2.89	3.673 (2)	141
N42—H7*N*···O3	0.88	2.08	2.957 (3)	173
N42—H8*N*···S1^xii^	0.88	2.88	3.749 (2)	169

Symmetry codes: (i) *x*+1, *y*, *z*; (ii) −*x*+2, −*y*+1, −*z*+1; (iii) *x*, *y*−1, *z*; (iv) *x*−1, *y*, *z*; (v) −*x*, −*y*+2, −*z*; (vi) −*x*+1, −*y*+2, −*z*; (vii) *x*+1, *y*+1, *z*; (viii) −*x*+1, −*y*+1, −*z*; (ix) −*x*+1, −*y*+1, −*z*+1; (x) −*x*+1, −*y*+2, −*z*+1; (xi) −*x*+2, −*y*+2, −*z*+1; (xii) −*x*, −*y*+1, −*z*.
