# Supplementary material for: Crystal structures of (aceto­nitrile-κN)tris­(pyridine-4-thio­amide-κN)bis­(thio­cyanate-κN)cobalt(II) aceto­nitrile disolvate and tetra­kis­(pyridine-4-thio­amide-κN)bis­(thio­cyanate-κN)nickel(II) methanol penta­solvate
Source: Acta Crystallogr E Crystallogr Commun. 2018 Jun 12;74(Pt 7):964–9. doi: 10.1107/S2056989018007612 (PMC6038621; doi:10.1107/S2056989018007612)
Supplement: Supplementary file 4 [file e-74-00964-sup4.pdf]

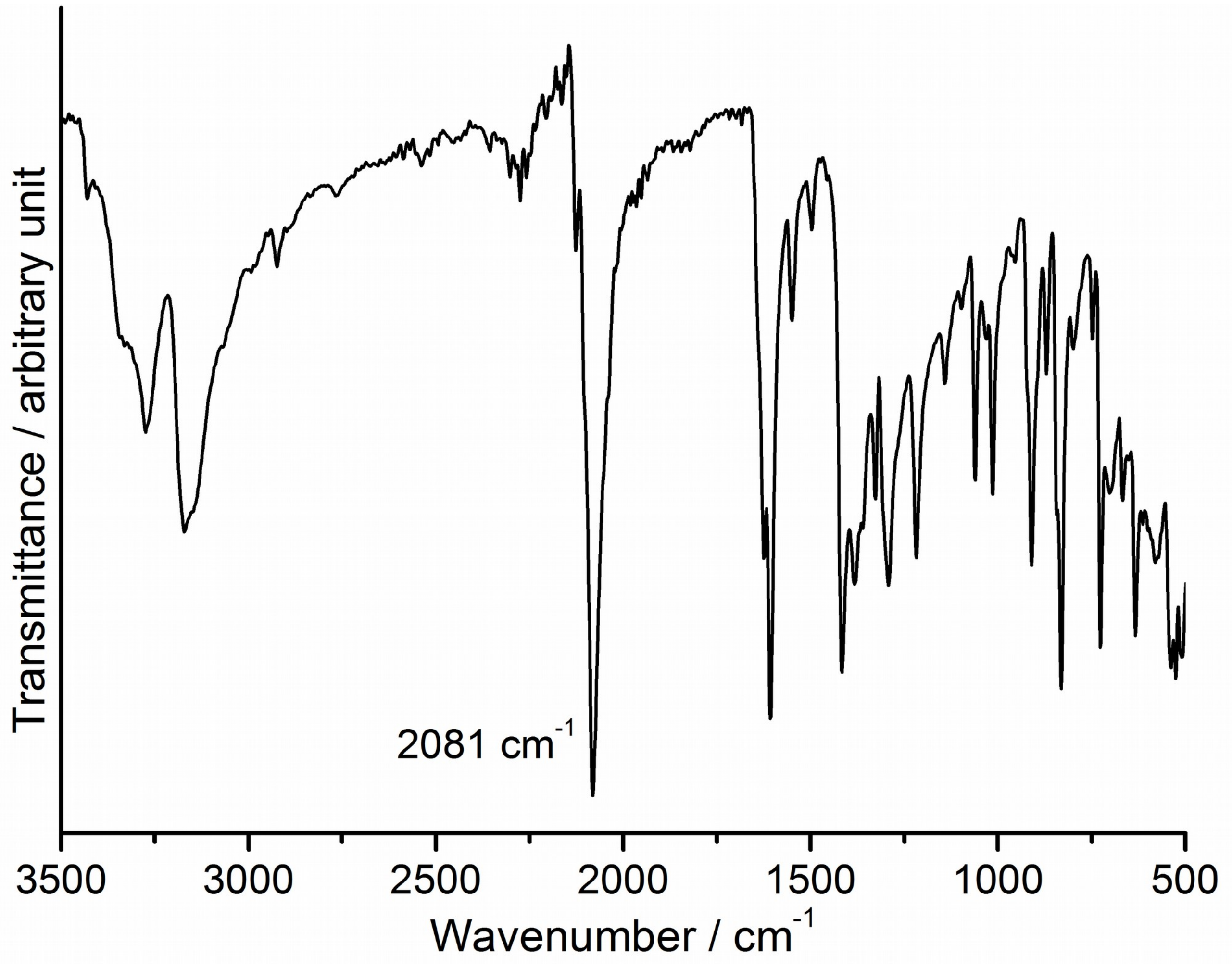

Fig. S1. IR spectra of **1**, measured from crystals selected by hand. Given is the value of the CN stretching vibration.

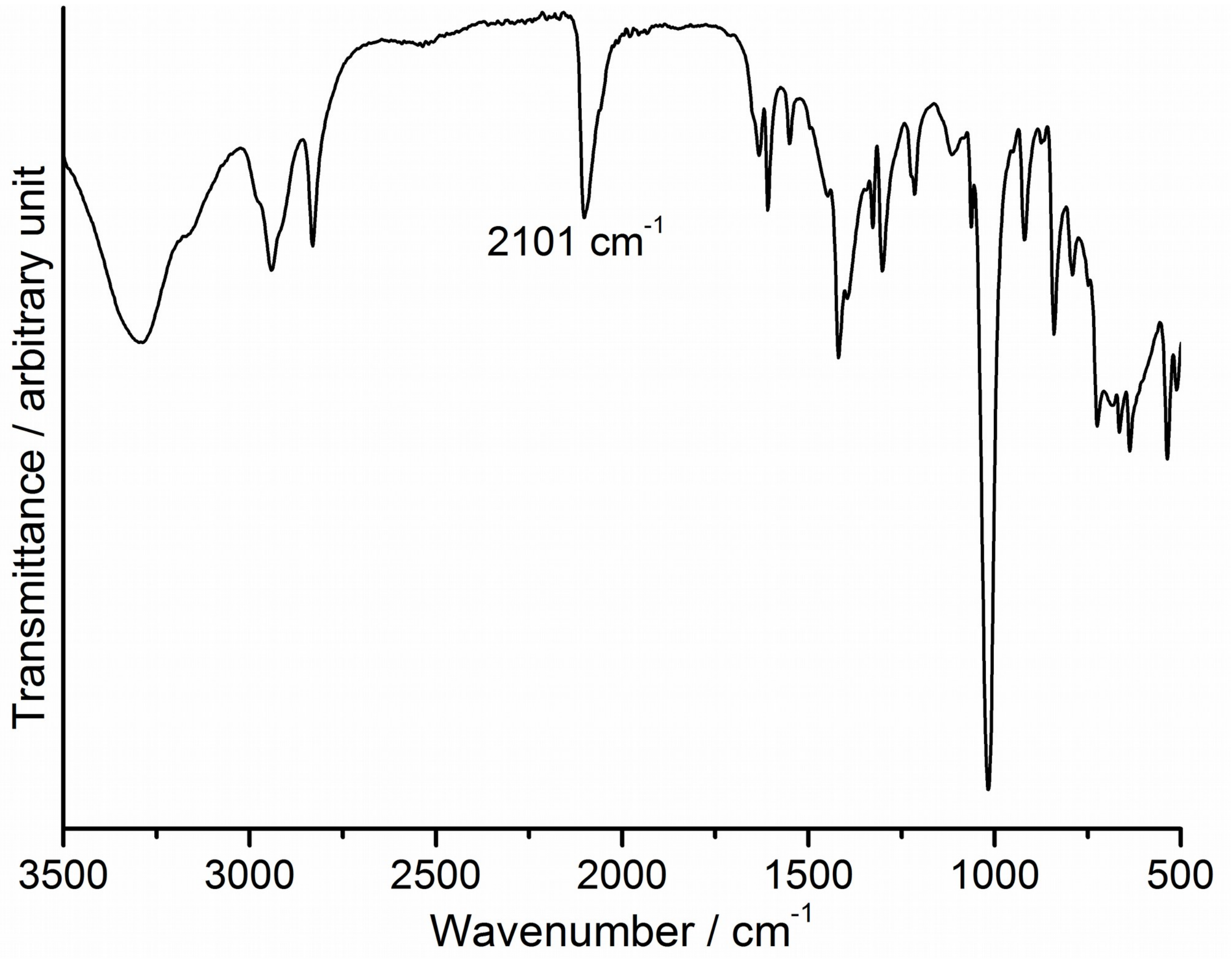

Fig. S2. IR spectra of **2**, measured from crystals selected by hand. Given is the value of the CN stretching vibration.
